# Adverse outcomes following hospitalization in acutely ill older patients

**DOI:** 10.1186/1471-2318-8-10

**Published:** 2008-05-14

**Authors:** Roger Y Wong, William C Miller

**Affiliations:** 1 Division of Geriatric Medicine, Department of Medicine, University of British Columbia, Canada; 2 Department of Occupational Science and Occupational Therapy, University of British Columbia, Canada; 3 GF Strong Rehabilitation Research Laboratory, Vancouver, British Columbia, Canada; 4 Vancouver Coastal Health Research Institute, Vancouver, British Columbia, Canada

## Abstract

**Background:**

The longitudinal outcomes of patients admitted to acute care for elders units (ACE) are mixed. We studied the associations between socio-demographic and functional measures with hospital length of stay (LOS), and which variables predicted adverse events (non-independent living, readmission, death) 3 and 6 months later.

**Methods:**

Prospective cohort study of community-living, medical patients age 75 or over admitted to ACE at a teaching hospital.

**Results:**

The population included 147 subjects, median LOS of 9 days (interquartile range 5–15 days). All returned home/community after hospitalization. Just prior to discharge, baseline timed up and go test (TUG, P < 0.001), bipedal stance balance (P = 0.001), and clinical frailty scale scores (P = 0.02) predicted LOS, with TUG as the only independent predictor (P < 0.001) in multiple regression analysis. By 3 months, 59.9% of subjects remained free of an adverse event, and by 6 months, 49.0% were event free. The 3 and 6-month mortality was 10.2% and 12.9% respectively. Almost one-third of subjects had developed an adverse event by 6 months, with the highest risk within the first 3 months post discharge. An abnormal TUG score was associated with increased adjusted hazard ratio [HR] 1.28, 95% confidence interval [CI] 1.03 to 1.59, P = 0.03. A higher FMMSE score (adjusted HR 0.89, 95% CI 0.82 to 0.96, P = 0.003) and independent living before hospitalization (adjusted HR 0.42, 95% CI 0.21 to 0.84, P = 0.01) were associated with reduced risk of adverse outcome.

**Conclusion:**

Some ACE patients demonstrate further functional decline following hospitalization, resulting in loss of independence, repeat hospitalization, or death. Abnormal TUG is associated with prolonged LOS and future adverse outcomes.

## Background

Acute care for elders units (ACE) focus on early rehabilitation, discharge planning, and delivering functionally oriented, patient-centered care [1,2]. ACE can improve outcomes [3,4], although some patients who are physically independent or terminally ill are less likely to benefit [5]. Since the publication of the original randomized trial [3], implementation of ACE has not been widespread [6,7]. Possible explanations include financial costs [6], mixed findings from subsequent studies [8-11], difficulty in predicting outcomes, and a lack of data on whether any benefit is sustainable. The prediction of ACE outcomes is influenced by multiple factors [12-15]. Recently, a systematic review showed that physical function, illness severity, cognition, comorbidity, presenting medical diagnosis, multiple medication use, and age could affect hospital length of stay (LOS), readmissions, discharge destination and mortality [16]. However, these findings have not been reproduced in prospective studies. In most hospitals, ACE patients are selected by age [3], although there may be benefits to selecting patients based on pre-morbid functional status. It remains unclear whether any of the commonly used clinical parameters, such as mobility and balance scores, cognitive and depression scores, illness severity and comorbidity scores, and clinical frailty scores, are useful in predicting the outcome of ACE patients. Ideally some of these standardized measurements might proactively identify individuals in ACE who are at risk for prolonged hospitalization, frequent hospital readmission, loss of independent residence following hospitalization, and death, so that specific strategies to optimize ACE outcomes can be targeted.

The overall goal of this article is to describe the outcomes of a population of older patients admitted to ACE by 3 to 6 months post discharge. Specifically, we report on the associations between socio-demographic and performance-based variables in mobility, balance, cognition, depression and activities of daily living (ADL) function at discharge with LOS during hospitalization (primary outcome), and which variables best predict mortality, readmission to acute care, and living disposition at 3 and 6 months after hospital discharge (secondary outcomes).

## Methods

### Setting

This is a prospective cohort study that recruited a sample of older adults admitted to 2 ACE units at the Vancouver General Hospital, which comprise 44 medical beds under the care of either internal medicine (clinical teaching units) or family medicine hospitalists. Detailed description of ACE was previously described [17]. We received approval from the institutional research ethics board to conduct this study.

### Subjects

We included ACE patients who consented to participate. They were eligible if they were 75 years or over (our hospital used this age cut-off as a surrogate marker to determine ACE eligibility), lived in the community pre-hospitalization, and could comprehend simple three-step commands in English. We also included ACE patients who transitioned through a separate sub-acute medical (SAM) unit after their initial stay in ACE. The SAM unit comprises of 32 medical beds for patients whose acute illnesses have stabilized but require time for functional recovery before returning home. We recognize the heterogeneity between ACE and SAM patients, but decided to include the ACE-SAM patients because they represented a common hospital trajectory post ACE stay, while increasing our sample size and power of the study. We did not include subjects if they were transferred from/to critical care or palliative care because these populations are not normally serviced by ACE; residing at a long term care facility prior to hospitalization; residing outside the catchment of the hospital (greater than 100 km distance); or deemed medically unstable by 1 of 2 internal medicine residents who reviewed each subject's physiologic parameters prior to any performance-based measure.

We calculated a priori the required sample size to be 150, using an alpha = 0.01, beta = 0.20 and over sampling for 20% attrition, which enabled modelling of up to 10 independent variables including interaction terms [18] at what Cohen [19] defined as a moderate to large effect size. To recruit 150 eligible subjects who would consent, we ended up screening ACE patients between October 2004 and October 2005 until the target sample size was reached. Three subjects declined to continue before baseline testing, therefore leaving 147 subjects from whom we collected baseline data (Table 1). The ACE subjects in this study were representative of the typical ACE population who survived hospitalization, at least based on age, sex, medical diagnosis, medication number and mobility independence when compared to the previously reported consecutive patient series from our ACE [10]. In the current cohort, there was no age and sex difference between study subjects and non-participants. Unfortunately we did not have authorized access to other baseline data on the non-participants.

**Table 1:** Characteristics of study subjects at baseline, 3-month and 6-month follow up.

**Characteristic**	**Baseline**	**3-month follow up**	**6-month follow up**
	**N = 147**	**N = 88**	**N = 72**

Age in years	83.9 ± 5.7	83.3 ± 5.2	83.3 ± 5.2
Female sex (%)	78 (53.1)	41 (46.6)	36 (50.0)
Marital status (%)			
Married	54 (36.7)	35 (39.8)	27 (37.5)
Widowed	67 (45.6)	38 (43.2)	34 (47.2)
Single	26 (17.7)	15 (17.1)	11 (15.3)
Residence before hospitalization (%)			
Community independent living	59 (40.1)	43 (48.9)	36 (50.0)
Other	88 (59.9)	45 (51.1)	36 (50.0)
Medical diagnosis (%)			
Cardiac disease	21 (14.3)	13 (14.8)	11 (15.3)
Pulmonary disease	4 (2.7)	4 (4.6)	2 (2.8)
Gastrointestinal disease	31 (21.1)	21 (23.9)	18 (25.0)
Infection	33 (22.5)	20 (22.7)	17 (23.6)
Neurologic disease	15 (10.2)	7 (8.0)	5 (6.9)
Diabetes mellitus	2 (1.4)	1 (1.1)	1 (1.4)
Functional decline	5 (3.4)	3 (3.4)	2 (2.8)
Cancer	3 (2.0)	0 (0.0)	0 (0.0)
Other	32 (21.8)	18 (20.5)	15 (20.8)
Number of prescription medications at admission	4.9 ± 3.0	4.7 ± 2.8	4.6 ± 2.8
Cumulative illness rating scale score	23.9 ± 4.2	23.9 ± 4.2	23.5 ± 4.2
Geriatric prognostic index score	1.7 ± 2.2	1.8 ± 2.0	1.7 ± 2.0
Clinical frailty scale score	4.7 ± 0.8	5.1 ± 0.9	4.8 ± 1.0
Number of independent ADL	4.8 ± 0.5	4.8 ± 0.5	4.9 ± 0.4
Folstein mini-mental state examination score	25.5 ± 3.9	26.2 ± 3.2	26.7 ± 2.8
Geriatric depression scale score	3.4 ± 2.6	3.2 ± 2.5	3.0 ± 2.5
Distance travelled in 2-minute walk test (meters)	62.9 ± 31.6	69.4 ± 33.8	73.1 ± 33.5
Time to complete timed up and go test (seconds)	31.6 ± 22.8	28.3 ± 20.5	26.6 ± 18.1
Sitting to standing balance test score	0.8 ± 1.1	0.8 ± 1.1	0.8 ± 1.1
Standing with 2 feet together balance test score	2.4 ± 1.2	2.5 ± 1.2	2.6 ± 1.2
Standing on 1 leg balance test score	1.2 ± 1.2	1.3 ± 1.3	1.4 ± 1.3

Plus-minus values are mean ± SD. Categorical data are reported as number of subjects and percentage in parentheses.

### Data collection and outcomes

All data was collected in an unblinded fashion. We pre-screened consecutive admissions to ACE and identified potential subjects. Informed consent was obtained from subjects, all of whom were capable of granting consent. We used uniform definitions and obtained the following baseline data from the hospital health records: socio-demographic data, medical diagnosis as defined by pre-established categories, and number of prescription medications at admission. We used the cumulative illness rating scale (CIRS) to measure medical complexity and comorbidity (score range 0–56), with higher scores indicating greater disease burden [20]; the geriatric prognostic index (GPI) to estimate the 1-year mortality risk after hospital discharge (score range 0–26), with higher scores predictive of higher mortality risk [21]; and the clinical frailty scale (CFS) to estimate the degree of fitness and frailty (score range 1–7), with higher scores indicating more severe frailty [22]. We also counted the number of independent ADL among 5 activities (eating, continence and/or functional ability to toilet, dressing, transferring, and bathing) prior to hospitalization.

We selected a number of validated performance-based tests that could be implemented without adding substantial burden to patients or workload to clinicians. Subjects underwent baseline testing in ACE just prior to anticipated discharge (median 2 days before discharge). We opted to obtain the functional measures just before discharge instead of at admission to allow for clinical stabilization of the patients, and to study how function could predict future adverse outcomes post hospitalization. Testing began with the 2-minute walk test (2MWT) to assess walking endurance and as a proxy of community ambulation potential [23], followed by a rest station during which the Folstein mini-mental state examination (FMMSE) was done to screen for cognitive impairment [24]. The timed up and go test (TUG) was then completed to assess walking skill/speed [23], followed by another rest station when the short form geriatric depression scale (GDS) was done to screen for major depression [25]. Testing finished with a battery of balance tests of lower extremity function (sitting to standing, standing with 2 feet together, standing on 1 leg, standing in a semi-tandem position, and standing in a full tandem position) that have been shown to be predictive of subsequent disability, nursing home admissions and mortality [26]. All tests were explained and demonstrated to the subjects, and conducted according to standardized protocols. Subjects who demonstrated excessive fatigue were offered the possibility to terminate or interrupt testing, with the opportunity to resume at a later time or on another day. No one requested this opportunity.

We scheduled the 3 and 6 months follow up home visits for all subjects at the time of hospital discharge providing reminders by mail one week and a telephone call 3 days prior to the actual visits. During each home visit, we conducted a structured personal interview to collect self-reported information on the current living disposition and the number of hospital readmissions in the time elapsed since the last point of data collection. A readmission was defined as a return to any hospital for at least 1 overnight stay. Subjects were allowed as much time as necessary to complete the interview, and could interrupt testing at any time if rest was needed.

The primary study outcome was LOS during hospitalization, defined as the number of hospital days spent during an entire hospital encounter based on hospital records. The secondary outcomes were self-reported living disposition, readmissions to any hospital since the index hospitalization in ACE, and death at 3 and/or 6 months post discharge. In addition, we defined an adverse event (a composite outcome) as non-independent living after hospitalization (that is, any living arrangement other than living in own or rental home), hospital readmission, or death at follow up.

### Statistical analysis

Descriptive statistics were computed for all variables: median and interquartile range for LOS, means and standard deviations for other continuous variables, and frequencies and percentages for categorical variables. We used multiple linear regression modeling to estimate the effect of variables on the primary outcome of LOS. The independent variables entered into the model included demographic variables, namely age, sex, marital status, independent living before hospitalization, clinical measures, such as CIRS, GPI, CFS at baseline, number of medications taken, number of independent ADL pre-admission, and functional measures including FMMSE scores, GDS scores, TUG scores, and the balance test scores while standing with 2 feet together. To satisfy normality assumptions, a logarithmic transformation was used. Standardized regression coefficients, their 95% confidence intervals and P values were reported. For the purpose of modeling, living disposition was collapsed into community independent living versus others. Due to co-linearity between independent variables, TUG was entered into the model instead of 2MWT, the standing with 2 feet together score was the only one entered among the balance tests performed, and standardized indices of CRIS, GPI and CFS were entered into the model instead of medical diagnoses because the former 3 were composite measures. While the TUG and 2MWT are highly correlated, we selected the TUG because it was performed more often in the current sample and also most highly correlated with the outcome on a bivariate level. Medical instability was not entered as a separate independent variable because of overlap with CIRS and GPI. The independent variables were entered into the regression model in a stepwise fashion, and the same results were obtained from stepwise, forward, and backward regression approaches.

In analyzing the secondary outcomes, the probabilities of any adverse event by 3 and 6 months were calculated using Kaplan-Meier survival analysis. A discrete-time Cox proportional-hazards model was used to estimate the unadjusted and adjusted hazard ratios for the composite adverse outcome. The same independent variables from the multiple linear regression analysis were used, except we also included CFS at 3 months as a time-dependent variable. Hazard ratios, their 95% confidence intervals, and P values are reported. Patients who declined to continue or could not be contacted were considered right-censored, assuming their follow-up times would otherwise be longer, and all contributed to time at risk in the Cox regression analysis. We accepted a level of significance when P < 0.05 for all analyses. All data analysis was completed using SAS software, Version 9.1 of the SAS System for Windows, SAS Institute Incorporation, Cary, NC, USA.

## Results

**Figure 1:**
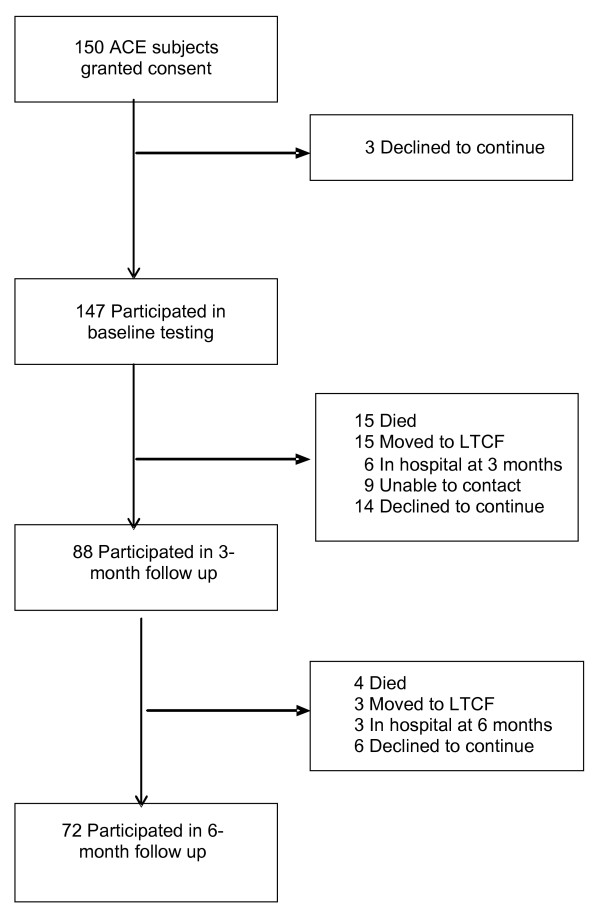
**Study flow diagram.** Of the original 150 subjects recruited, 88 participated at the 3-month and 72 participated at the 6-month follow up after discharge from ACE. LTCF = long term care facility.

Of the 147 ACE patients who had baseline data, 88 subjects (59.9%) who remained in the study by 3 months had not developed an adverse event of non-independent living, readmission or death, while 72 (49.0%) were free of an adverse event by 6 months (Figure 1). There were 46 subjects (31.3%) who had developed an adverse event by 6 months (see below), and 29 subjects (19.7%) either declined to continue or could not be contacted. These subjects did not differ in terms of demographics from the rest of the sample. If subjects moved to a long term care facility, they were not interviewed as they were considered to have experienced an adverse outcome. The characteristics of our subjects at each study time point are summarized in Table 1. Of note, all 72 subjects who participated at 6 months were included in the 3-month follow-up. The population seen at 6 months was similar to those who did not follow up, except the former had higher FMMSE (26.7 ± 2.8 vs. 24.2 ± 4.4, P < 0.0001), faster TUG (26.6 ± 18.1 vs. 36.3 ± 25.8, P = 0.009), and shorter LOS (10.6 ± 7.9 vs. 16.9 ± 20.9, P = 0.017).

Data on the primary outcome (LOS) was available for all subjects (N = 147), who spent a median time of 9 days (interquartile range 5–15 days). We used simple linear regression to estimate the standardized regression coefficients of various characteristics that influenced LOS (Table 2). Specifically, 3 characteristics resulted in significant bivariable associations: TUG (P < 0.001), balance test score while standing with both feet together (P = 0.001), and CFS score at baseline (P = 0.02). However, in multiple regression analysis, TUG was the only independent variable that was significantly associated with LOS (standardized regression coefficient 0.33, 95% confidence interval (95% CI) 0.16 to 0.49, P < 0.001).

**Table 2:** Linear regression analyses to estimate the standardized regression coefficients of various characteristics on hospital length of stay (the primary outcome).

**Characteristic**	**Standardized regression coefficient (95% CI)**	**P Value**
Age	0.03 (-0.14, 0.20)	0.73
Male sex	0.05 (-0.13, 0.22)	0.60
Married	0.06 (-0.29, 0.41)	0.75
Independent living before hospitalization	0.10 (-0.07, 0.27)	0.24
Number of medications at admission	-0.14 (-0.31, 0.03)	0.10
Cumulative illness rating scale score	0.08 (-0.09, 0.25)	0.37
Geriatric prognostic index score	-0.06 (-0.23, 0.11)	0.52
Clinical frailty scale score	0.20 (0.04, 0.37)	0.02
Number of independent ADL	0.06 (-0.11, 0.23)	0.46
Folstein mini-mental state examination score	0.01 (-0.16, 0.18)	0.94
Geriatric depression scale score	0.06 (-0.12, 0.23)	0.52
Timed up and go test score	0.33 (0.16, 0.49)	<0.001
Standing with 2 feet balance test score	-0.28 (-0.44, -0.12)	0.001

Characteristics with positive coefficients imply increased values were associated with longer length of stay, whereas negative coefficients imply vice versa. CI = confidence interval.

**Figure 2:**
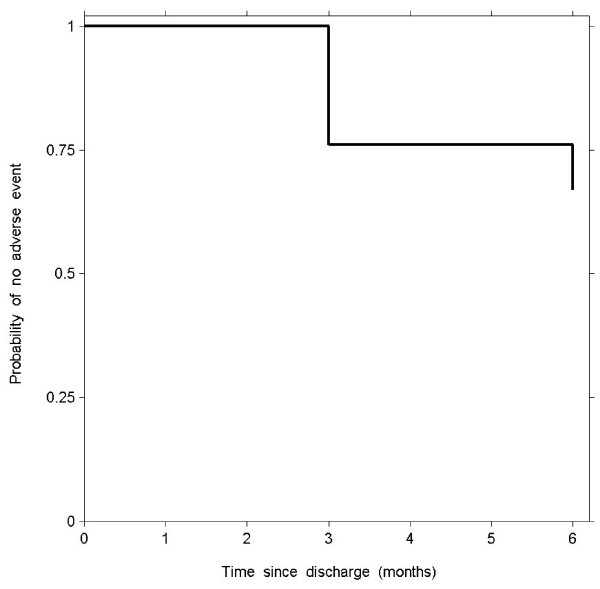
**Kaplan-Meier survival curve at 3 months and 6 months.** The estimated survival probability is 0.76 and 0.67 respectively.

The probability of an adverse event occurring within the first 3 and 6 months post discharge was shown in the Kaplan-Meier survival analysis (Figure 2). The unadjusted hazard ratios of various risk factors for an adverse event using the Cox proportional-hazards model are shown in Table 3. Higher FMMSE score was associated with reduced hazard, whereas longer TUG time and older age were associated with increased hazard for an adverse event. In multiple Cox proportional-hazards analysis higher FMMSE score (adjusted HR 0.89, 95% CI 0.82 to 0.96, P = 0.003) and independent living before hospitalization (adjusted HR 0.42, 95% CI 0.21 to 0.84, P = 0.01) were associated with reduced hazard, whereas longer TUG times led to increased hazard ratios (adjusted HR 1.28, 95% CI 1.03 to 1.59, P = 0.03 for 20 seconds longer).

**Table 3:** Risk factors and the associated unadjusted hazard ratios for an adverse event (defined as non-independent living, hospital readmission, or death at follow up) using the Cox proportional-hazards model.

**Characteristic**	**Hazard ratio (95% CI)**	**P Value**
Age (years)	1.05 (1.00, 1.11)	0.05
Male sex	0.97 (0.54, 1.74)	0.92
Married	0.89 (0.48, 1.63)	0.69
Independent living before hospitalization	0.56 (0.30, 1.05)	0.07
Number of medications at admission	0.99 (0.89, 1.09)	0.80
Higher cumulative illness rating scale score	1.03 (0.96, 1.10)	0.46
Higher geriatric prognostic index score	1.01 (0.89, 1.16)	0.86
Higher clinical frailty scale score at baseline	1.25 (0.88, 1.79)	0.22
Higher clinical frailty scale score at 3 months	1.25 (0.59, 2.64)	0.56
More independent ADL	0.92 (0.52, 1.64)	0.78
Higher mini-mental state examination score	0.91 (0.85, 0.98)	0.01
Higher geriatric depression scale score	1.08 (0.98, 1.20)	0.14
Time to complete timed up and go test (seconds)*	1.28 (1.03, 1.59)	0.03
Higher balance test score while standing with 2 feet	0.86 (0.67, 1.10)	0.23

* The hazard ratio associated with the timed up and go test is for a 20 second increase.

CI = confidence interval.

In addition, we added LOS to the Cox regression analysis on adverse events. The hazard of an adverse event increased by 4% for each additional day in hospital (unadjusted HR 1.04, 95% CI 1.02 to 1.06, P < 0.0001). After re-running the multiple Cox proportional-hazards analysis, LOS replaces TUG. This final model (Table 4) shows higher FMMSE score (adjusted HR 0.87, 95% CI 0.80 to 0.95, P = 0.0011) and independent living before hospitalization (adjusted HR 0.34, 95% CI 0.15 to 0.74, P = 0.0063) were associated with reduced hazard, whereas longer LOS led to increased hazard ratios (adjusted HR 1.06, 95% CI 1.03 to 1.09, P < 0.0001).

**Table 4:** Statistically significant risk factors and the associated adjusted hazard ratios for an adverse event (defined as non-independent living, hospital readmission, or death at follow up) using the multiple Cox proportional-hazards model.

**Characteristic**	**Hazard ratio (95% CI)**	**P Value**
Higher mini-mental state examination score	0.87 (0.80, 0.95)	0.0011
Independent living before hospitalization	0.34 (0.15, 0.74)	0.0063
Longer length of stay in hospital	1.06 (1.03, 1.09)	<0.0001

CI = confidence interval.

## Discussion

In this prospective cohort study we followed older patients admitted to ACE longitudinally for 6 months post discharge. Of this sample 59.9% remained free of an adverse event of non-independent living, readmission or death by 3 months, and 49.0% were still event free by 6 months. The 3 and 6-month mortality rate was 10.2% and 12.9% respectively. Although all subjects were initially able to return home, almost one-third had developed an adverse event by 6 months, with the highest probability of an adverse event occurring within the first 3 months. An abnormal TUG score was associated with increased risk of an adverse event, whereas a higher FMMSE score and independent living before hospitalization were associated with reduced risk. The baseline TUG, bipedal stance balance test, and CFS scores showed significant associations with LOS during hospitalization, with the TUG score as the only independent predictor in multiple regression analysis.

Our study captured ACE patients who survived hospitalization and were able to return to independent living at discharge. Their CFS scores put them in the mildly frail category. We did not capture the moderately or severely frail patients, who were ineligible or did not consent. We recognize a number of our study subjects could not be followed up by 3 and 6 months due to an adverse event defined as non-independent living, readmission or death. We used a composite adverse event rather than looking at each adverse event separately due to relatively small numbers of events. While our finding might be intuitive for a moderately or severely frail group, it is nonetheless intriguing to see this substantial dropout rate for our mildly frail cohort.

Our findings extend and support the literature on the outcomes and their predictors in older patients following admission to ACE. Beyond the immediate benefits of ACE care [3,8,10], ACE patients are at risk for adverse outcomes, especially in the immediate 3 months after hospitalization. This is unlikely due to premature discharge during the index hospital stay, for the mean LOS in this sample is actually higher than other published data [10]. Nor is this likely an independent effect of age, sex, marital status, comorbidity, polypharmacy or depression based on our analysis, although we cannot exclude the possibility that other important confounding variables could be missing. Rather it likely reflects the overall frailty of ACE patients (clinical frailty scale scores 4.7 ± 0.8 at baseline, 5.1 ± 0.9 at 3 months, and 4.8 ± 1.0 at 6 months). Measures of physical function have shown to be predictive of outcomes in hospitalized older adults [27-29], and in particular, mobility has been found to be predictive of ADL function [30]. The TUG is a proxy of household mobility and has been found to be reliable and valid in a variety of older populations [31,32]. In addition, previous studies on geriatric evaluation and management units have long established that pre-morbid function was one of the key predictors for hospital outcomes [33], at least within the setting of highly selective patients who were neither too well nor too frail to benefit. Since our ACE took "all comers" age 75 or over (our hospital made this operational decision deliberately, not to violate the fundamental precept of targeting appropriate patients, but rather for quality improvement and ethical reasons so that no older adults would be excluded from receiving ACE care which we considered best clinical practice), a functional measure like TUG would likely be predictive of future outcomes. We observe that the TUG alone accounted for 11% of the variance in the multiple regression model for LOS (that is, R-squared 0.11), and was the highest and only statistically significant contributor among the other 12 independent variables selected in our attempt to predict LOS. This has important implications, for the TUG is easy to do, inexpensive, and does not require extensive training or special equipment. The other predictors of LOS (standardized balance test with 2 feet standing together, the CFS) and predictors of adverse event (the FMMSE, self reported residence before hospitalization), also have potential for easy implementation clinically. The psychometric properties of the performance based measures are well established in the literature, therefore allowing uniform interpretation across different ACE units. We recognize that other non-clinical, non-functional factors might impact on LOS, such as social work availability for discharge planning, day of admission during the week, etc. The first 3 months following discharge from ACE represents a high-risk period of non-independent living, readmission or death, and the risk thereafter appears to taper somewhat. This raises the question of whether delivering timely post-discharge interventions within this period will reduce or even eliminate this risk. The nature of such hospital-based outreach interventions has not been well defined, although there is some evidence that home programs might improve outcomes [34,35]. Further studies are warranted.

Our findings should be interpreted within the context of their limitations. The results might not be generalizable to all older adults admitted to hospital, such as those from nursing homes or who speak minimal/no English. ACE Patients who consented in our study were likely more fit and healthy as compared with the majority of those who did not/could not consent, and therefore might not be representative of the main ACE population. While subgroup analyses to identify the best predictors in males and females might be interesting, unfortunately our study was not adequately powered to do so. Our subjects were generally cognitively intact, and we recognize that functional attributes of cognitively impaired individuals might be different and need to be studied separately. Specifically it would be helpful to identify delirium cases and their impact in a future study. Hindsight would suggest exclusion of SAM patients from the analysis in an attempt to reduce heterogeneity, although this would imply screening for more ACE patients before recruiting the required number, thereby raising question on the representation of the cohort. There might be seasonal effect on the type of ACE patients and outcomes since the study was conducted from July to January, and findings might differ if the study was done during the winter period. Although we have made efforts within the available resources to extend the follow up duration in this study to 6 months post discharge, ideally the frequency and length of follow up should be greater. Our follow up duration nonetheless exceeds current published knowledge on ACE patients and contributes to our understanding of this population. We acknowledge the attrition of the initial patient cohort could make the interpretation of the secondary objective findings (predicting functional decline) challenging. We lost 19.7% of our subjects to follow up, which matched with our original 20% over-sampling to prevent type-2 error. The dropouts should not affect the analysis on LOS as all independent variables included were measured at baseline. Furthermore, dropouts still contributed to time at risk for an adverse event. Finally, we did not conduct the regression analyses on another independent group of patients to assess the validity of the analyses.

## Conclusion

For many older adults discharged from ACE programs, independent functioning at home and in the community is of major concern. The challenges faced by these individuals do not end once their acute medical problem is addressed. In fact, many experience significant functional decline during and following hospitalization, which in turn leads to loss of independence (including institutionalization), repeat hospitalization, or death. Our study findings identify TUG as potentially useful for identifying acutely ill elderly patients who are at risk of adverse outcomes after hospitalization in a selected sample of ACE patients who are able to return to independent living after discharge. The goal of this work is to develop foundational data for interventions that may influence functional impairments and alter future outcomes, although the exact nature of such interventions is yet to be determined and requires further studies.

## Competing interests

The authors declare that they have no competing interests.

## Authors' contributions

Both authors contributed to the development of the conceptualization and design of the study. RYW was primarily responsible supervising the data analyses and interpreting and preparing the manuscript. WCM supervised the data collection and provided assistance with data analyses and editing the final manuscript. All authors read and approved the final draft of the manuscript.

## Pre-publication history

The pre-publication history for this paper can be accessed here:


